# Evaluation of an Antioxidant and Anti-inflammatory Cocktail Against Human Hypoactivity-Induced Skeletal Muscle Deconditioning

**DOI:** 10.3389/fphys.2020.00071

**Published:** 2020-02-12

**Authors:** Coralie Arc-Chagnaud, Guillaume Py, Théo Fovet, Rémi Roumanille, Rémi Demangel, Allan F. Pagano, Pierre Delobel, Stéphane Blanc, Bernard J. Jasmin, Dieter Blottner, Michele Salanova, Mari-Carmen Gomez-Cabrera, José Viña, Thomas Brioche, Angèle Chopard

**Affiliations:** ^1^DMEM, Université Montpellier, INRAE, Montpellier, France; ^2^Freshage Research Group, Department of Physiology, Faculty of Medicine, CIBERFES, Fundación Investigación Hospital Clínico Universitario/INCLIVA, University of Valencia, Valencia, Spain; ^3^Faculté des Sciences du Sport, Mitochondries, Stress Oxydant et Protection Musculaire, Université de Strasbourg, Strasbourg, France; ^4^Department of Cellular and Molecular Medicine and Centre for Neuromuscular Disease, Faculty of Medicine, University of Ottawa, Ottawa, ON, Canada; ^5^IPHC, CNRS, Université de Strasbourg, Strasbourg, France; ^6^Berlin Center for Space Medicine, Integrative Neuroanatomy, Charité Universitätsmedizin Berlin, Berlin, Germany

**Keywords:** muscle wasting, inactivity, oxidative stress, antioxidants, cell signaling

## Abstract

Understanding the molecular pathways involved in the loss of skeletal muscle mass and function induced by muscle disuse is a crucial issue in the context of spaceflight as well as in the clinical field, and development of efficient countermeasures is needed. Recent studies have reported the importance of redox balance dysregulation as a major mechanism leading to muscle wasting. Our study aimed to evaluate the effects of an antioxidant/anti-inflammatory cocktail (741 mg of polyphenols, 138 mg of vitamin E, 80 μg of selenium, and 2.1 g of omega-3) in the prevention of muscle deconditioning induced by long-term inactivity. The study consisted of 60 days of hypoactivity using the head-down bed rest (HDBR) model. Twenty healthy men were recruited; half of them received a daily antioxidant/anti-inflammatory supplementation, whereas the other half received a placebo. Muscle biopsies were collected from the vastus lateralis muscles before and after bedrest and 10 days after remobilization. After 2 months of HDBR, all subjects presented muscle deconditioning characterized by a loss of muscle strength and an atrophy of muscle fibers, which was not prevented by cocktail supplementation. Our results regarding muscle oxidative damage, mitochondrial content, and protein balance actors refuted the potential protection of the cocktail during long-term inactivity and showed a disturbance of essential signaling pathways (protein balance and mitochondriogenesis) during the remobilization period. This study demonstrated the ineffectiveness of our cocktail supplementation and underlines the complexity of redox balance mechanisms. It raises interrogations regarding the appropriate nutritional intervention to fight against muscle deconditioning.

## Introduction

Skeletal muscle is a plastic tissue able to adapt to intrinsic and environmental stresses (Harridge, 2007). While physical exercise and training reinforce our muscles, in contrast, situations of hypoactivity such as immobilization, sedentary lifestyle, or microgravity environments lead to skeletal muscle deconditioning. In this context, space agencies must always work on the optimization of countermeasures, especially in astronaut training programs and supplementation to preserve their work capacity and health. Muscle deconditioning is the consequence of a dysregulation of muscle homeostasis that triggers structural and functional alterations (Jackman and Kandarian, 2004; Chopard et al., 2009a; Baldwin et al., 2013). It translates mainly to a loss of muscle mass and myofiber atrophy, largely induced by a dysregulation of protein balance signaling pathways (Kandarian and Stevenson, 2002; Glass, 2005; Degens and Alway, 2006; Ventadour and Attaix, 2006). Amyotrophy is also accompanied by a greater loss of strength and power (Brioche et al., 2016). Inactive skeletal muscles are also exposed to metabolic remodeling, affecting the myofiber typology and their contractile properties. Indeed, alteration of some actors of the oxidative system (mitochondria and enzymes) contributes to the shift toward a glycolytic profile to the detriment of the oxidative one (Widrick et al., 1999; Fitts et al., 2010).

Another important element influenced by hypoactivity situations is the redox balance. It consists of an equilibrium between pro-oxidant molecules and antioxidant defenses (Powers et al., 2016). In skeletal muscle, mitochondria, NADPH oxidase, and xanthine oxidase are the main sources of the reactive oxygen and nitrogen species (RONS) (Murphy, 2009; Gomez-Cabrera et al., 2013). The antioxidant defenses are composed of two systems, an endogenous one that relies on enzyme actions (superoxide dismutase, catalase, and glutathione peroxidase) and an exogenous one that relies on various actors (glutathione, vitamins C and E, polyphenols, and carotenoids). The steady state of the redox balance is essential for the healthy function of organisms. For example, energy production, cellular proliferation, gene expression, and immunity are some of the functions that it regulates (Sies, 2017). In prolonged muscle-disuse situations, this balance shifts in favor of pro-oxidants and triggers oxidative stress. It translates into a dysregulation of various signaling pathways in the cells, including those linked to protein balance, apoptosis, and regeneration or excitation-contraction coupling (Zerba et al., 1990; Powers et al., 2011a,b; Salanova et al., 2013). On the other hand, inflammatory processes play a role in RONS production and contribute to the alteration of protein balance pathways (Flohé et al., 1997; Li and Karin, 1999; Stamler and Meissner, 2001). In this context, microgravity and hypoactivity-induced high inflammation levels associated with oxidative stress accentuate the muscle deconditioning phenomenon (Reid et al., 1993; Powers et al., 2011b).

The head-down bed rest (HDBR) model is one of the most commonly used models in space physiology, as it mimics weightlessness conditions (Pavy-Le Traon et al., 2007). In addition to involving chronic hypoactivity and muscle unloading, the −6° tilt of the subjects induces fluid redistribution similar to that observed in spaceflight (Hargens and Vico, 2016). Nonetheless, apart from the aerospace context, HDBR represents a strong experimental model in the study of various situations linked to immobilization and reduced activity (sport field, clinical stay, etc.).

The present study relates to the “cocktail study” carried out under the aegis of the European Space Agency (ESA), with the aim of evaluating the effect of an antioxidant/anti-inflammatory cocktail as a countermeasure during a 2-month bedrest experiment. Considering previous knowledge about cellular alterations induced by inactivity, it is legitimate to think that limiting cellular oxidative stress and inflammation levels could be an appropriate strategy. The currently available literature highlights the interesting roles of some molecules in muscle deconditioning prevention. Omega-3 fatty acids, known for their anti-inflammatory action, could decrease atrophy and low-grade inflammation levels and activate the protein synthesis pathway during unloading situations (You et al., 2010; Jeromson et al., 2015). Moreover, in a model of cachexia in rodent cancer-induced cachexia, omega-3 fatty acid supplementation has shown a preventive on muscle atrophy, and data in elderly people showed that omega-3 fatty acids can activate the protein synthesis mTOR pathway (Smith et al., 2011a,b). In animals, vitamin E supplementation was able to decrease proteolysis and muscular atrophy (Servais et al., 2007). Vitamin E is also known for its anti-inflammatory properties through inhibition of the NF-kB pathway which is well described in disuse atrophy models to activate proteolytic pathways (Huey et al., 2008; Khor et al., 2014). Vitamin E is often coupled with Selenium as this latter scavenges RONS and boosts the intracellular effects of vitamin E. Combined with selenium oligo element, vitamin E administration in rats demonstrated positive effects on oxidative stress markers *via* the activation of the endogenous antioxidant system (Beytut et al., 2018). Vitamin E and selenium combination also showed beneficial effect in patients with facioscapulohumeral dystrophy (Passerieux et al., 2015). Polyphenols known for their antioxidant properties given as pure molecules such as quercetin, epigallocatechin 3, resveratrol or as natural plant extracts (green tea or grape seed extracts) showed beneficial effect on rodent muscle atrophy (Alway et al., 1985; Momken et al., 2011; Lambert et al., 2015; Meador et al., 2015; Mukai et al., 2016). For example, during mechanical unloading, Momken et al. (2011) tested a nutritional countermeasure based on resveratrol administration. Its administration in rats was able to maintain protein balance, soleus muscle mass, and maximal force contraction. While current data suggest that some bio-active compounds, taken alone or as natural extracts, enhance several aspects of muscle and whole-body metabolic control, a single micronutrient is unlikely to be strong enough to reverse the wide range of deleterious effects induced by physical inactivity. Recently, the notion of nutrient cocktails, to trigger additive and/or synergistic effects between bio-active compounds, has been proposed (Damiot et al., 2019). In this recent study, we examined the capacity of a nutrient cocktail composed of polyphenols, omega-3 fatty acids, vitamin E, and selenium to prevent the expected metabolic alterations induced by physical inactivity and sedentary behaviors. The cocktail used in the study of Damiot et al. fully prevented the hypertriglyceridemia, the drop in fasting HDL and total fat oxidation, and the increase in *de novo* lipogenesis. Moreover, the cocktail limited the decrease in type-IIa muscle fiber cross-sectional area and decreased protein ubiquitination content.

This human study is the first to focus on cocktail supplementation with antioxidant/anti-inflammatory molecules as a countermeasure to the deleterious effects of a prolonged simulated microgravity period, and our aim was to evaluate the effects of the oral supplement on muscle deconditioning.

## Materials and Methods

### Subjects and Ethics Statement

Twenty healthy, active (between 10,000 and 15,000 steps per day) males were selected for this experiment (age: 34 ± 8; height: 176 ± 5 cm; weight: 73.5 ± 6.1 kg; BMI: 23.7 ± 1.5). The subjects had no medical history or physical signs of neuromuscular disorders. They were nonsmokers and were not taking any drugs or medications. All subjects gave informed consent to the experimental procedures, which were approved by the local ethics committee (CPP Sud-Ouest et Outre-Mer I, France, number ID RCB: 2016-A00401–50) in accordance with the Declaration of Helsinki. All experiments were conducted at the Space Clinic of the Institute of Space Medicine and Physiology (Medes-IMPS, Rangueil Hospital) in Toulouse (France) and were sponsored by the European Space Agency (ESA) and the French National Space Agency (CNES).

### Overall Study Design

This experiment consisted of a 2-month HDBR period with a 14-day baseline data collection period before HDBR and a 14-day recovery period after it. During the 2-month HDBR period, the subjects laid in a supine position with a −6° tilt to preserve simulated microgravity effects. Participants were randomly assigned to two groups on a double-blind basis. Ten of the participants were part of the “Placebo” group, whereas the ten others were part of the “Cocktail” group and received a daily antioxidant/anti-inflammatory cocktail during the 2-month bedrest period. All pills were taken at mealtimes to reduce the risks of secondary effects affecting the gastrointestinal area. Each subject had a daily medical examination, and the MEDES team took several standardized measurements. Room lighting was on between 07.00 and 23.00 h. During the entire hospitalization phase, the diet was monitored, and the meals were defined by the MEDES nutritionist and provided by Toulouse Hospital. During the HDBR period, the subjects remained in a supine position with a −6° tilt continuously, even during a daily 20-min extraction for toilet procedures and weighing (to preserve as much as possible the effects of simulated microgravity) and were instructed not to produce any unnecessary movements with their limbs.

### Cocktail Composition

The nutrient cocktail composition was based on a previous study (Damiot et al., 2019). The daily dose administered was composed of a 741 mg of bioactive polyphenol compound mix (XXS-2A-BR2 mix, Spiral Company, Dijon, France), 138 mg of vitamin E coupled with 80 μg of selenium (Solgar, Marne la Vallée, France), and 2.1 g of omega-3 (Omacor, Pierre Fabre Laboratories, Toulouse France). The daily dose of polyphenols was exactly composed by flavonols (323.4 mg), phenylpropanoides (45.6 mg), oligostilbènes, (78.0 mg), acide hydroxycinnamiques (50.4 mg), flavanols (135.6 mg), and flavanones (108.0 mg) and consisted of six pills per day: two at breakfast, two at lunch and two at diner. As there is no Dietary References Intake (DRI) available for polyphenols, the ~700 mg/day dose was based on several reviews on the bioavailability and bioefficacy of polyphenols in humans and other studies that tested the effects of polyphenols on exercise performance and oxidative stress (Manach et al., 2005; Myburgh, 2014; Somerville et al., 2017; Damiot et al., 2019). Polyphenol nutrient cocktail derived from food sources that consist of Liliaceae, Vernenaceae, Lamiaceae, Vitaceae, Rubiaceae, Theaceae, and Rutaceae Genres consisting of *Allium cepa*, *Lippia citriodora*, *Ajuga reptans*, *Vitis vinifera*, *Coffea robusta*, *Camellia sinensis*, and *Citrus aurantium*. For vitamin E, a single pill was orally ingested per day with breakfast. The daily proposed dose was respectively 6 and 5 times lower than the maximum allowed doses according to the US National academy of sciences. The 3 g daily dose of omega-3 was based on French pharmacopeia recommendations for hypolipemic effects (2–4 g/day) and was provided as 1 pill per meal which is within the daily dose used in most clinical studies (Ng et al., 2014; Hodson et al., 2017). This daily dose thus corresponded to 1.1 g of eicosapentaenoic acid (EPA) and 1 g of docosahexaenoic acid (DHA).

### Measurements of Maximal Isometric Voluntary Contraction

To characterize the strength of the subjects’ lower limb muscles, the maximal voluntary isometric contraction (MVC) strength was measured using a ConTrex device. The left leg of the participants was used to determine strength in knee and ankle flexors and extensor muscle groups. MVC was determined at an angulation of 80° for the knee and 0° for the ankle compared to the standard referential.

**Figure 1:**
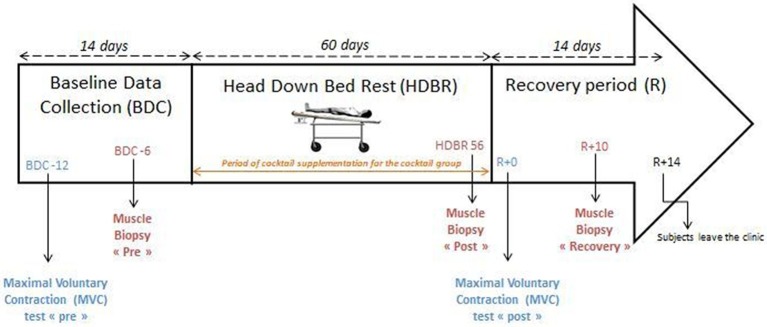
Representative time axis of the experiment.

Two measurements were completed, with the first one performed 12 days before the bed rest protocol (see Figure 1) and the second one just before the end of bed rest (day of restart of the upright posture). To carry out these tests, each participant was familiarized with the equipment and the standardized protocol before conducting the tests. During the assessments, participants were seated on the chair of the ConTrex device and firmly attached to it to avoid disturbance of movement.

The protocol was similar for each muscle group; after a short warm-up in neutral position, a series of measurements were recorded with a 30-s recovery interval. Each series consisted of a first extension movement followed by an isometric contraction and a flexion movement followed by an isometric contraction. Each one was maintained between 5 and 7 s, and 2 min of recovery was provided every 3 sets of measurements. The total duration of the test was 15 min per pair of agonist/antagonist muscle groups. To determine the MVC, the maximum strength level (N.m) achieved during the test was retained.

### Muscle Biopsy

Skeletal muscle biopsies were performed before (pre), at the end (post), and 10 days after the end of the HDBR period (see Figure 1) from the vastus lateralis muscle according to a well-established method using a 5 mm Bergström biopsy needle under sterile conditions and local anesthesia (1% lidocaine). The three biopsies were obtained from the same leg of each subject (the right leg) as near each other as possible because of potential anatomical variations. For each biopsy, one piece was immediately embedded in a small silicone cast filled with a cryoprotectant (OCT, Sakura Finetek), immediately frozen in liquid nitrogen cooled isopentane, and stored at −80°C until further histological analysis. The other piece was rapidly frozen in liquid nitrogen and stored at −80°C for protein content quantification.

### Cryosectioning and Immunohistochemistry

To determine cross-sectional area (CSA) and muscle fiber typing, transverse serial cross sections (10 μm thick) of vastus lateralis samples were obtained using a cryostat maintained at −25°C. The same methodology of Demangel et al. (2017) was used for CSA and MyHC (types I, II and IIa) labeling.

### Protein Isolation and Western Blotting

Muscle samples were exactly treated as in the study of Pagano et al. (2018). For western blots, we present cropped images of a representative subject of each group.

### Carbonylated Protein Determination

Determination of carbonylated protein levels was assessed by immunoblot detection of protein carbonyl groups using the “OxyBlot” protein oxidation kit (Millipore, MA, USA). Total protein carbonyls were quantified with the OxyBlot kit by densitometry of the blotting, relativized by the densitometry of the ponceau red staining of the membrane.

### Antibodies

Primary and secondary antibodies used for western blot and immunohistochemistry are presented in Table 1, with their respective references and dilutions used.

**Table 1:** List of primary and secondary antibodies, their reference, provider, and dilution.

Antibody	Reference	Commercial	Dilution
p-4EBP1	9451	Cell signaling	1:1,000
4EBP1	9644	Cell signaling	1:1,000
4-HNE	46545	Abcam	1:1,000
ATG7	8558	Cell signaling	1:1,000
Catalase	110704	Genetex	1:1,000
Citrate synthase	Sc-390693	Santa Cruz	1:200
COX IV	Sc-69360	Santa Cruz	1:200
Cytochrome c	Sc-13560	Santa Cruz	1:200
p-Eif2α	3398	Cell signaling	1:1,000
Eif2α	9722	Cell signaling	1:1,000
Gpx	3206	Cell signaling	1:1,000
LC3	L7543	Sigma	1:400
p-PRAS40	13175	Cell signaling	1:1,000
PRAS40	26915	Cell signaling	1:1,000
PGC1-α	AB3242	Millipore	1:1,000
p-ULK1	6888	Cell signaling	1:1,000
ULK1	4776	Cell signaling	1:1,000
Anti-MyHC1	BA-D5	DSHB	1:10
Anti-MyHC2	M4276	Sigma-Aldrich	1:200
Anti-MyHC2a	SC-71	DSHB	1:10
Anti-mouse - HRP	7076	Cell signaling	1:5,000
Anti-rabbit - HRP	7074	Cell signaling	1:5,000
Anti-goat - HRP	Sc-2953	Santa Cruz	1:4,000
Anti-rabbit - Alexa 488	A11034	Invitrogen	1:800
Anti-mouse - Alexa 588	A11031	Invitrogen	1:800

### Statistics

All values are expressed as the mean ± SEM, and the significance level was set at *p* < 0.05. The normal distribution of the samples was assessed by the Shapiro-Wilk test. To compare our two different groups and their different conditions (pre-post-recovery), we used a two-way repeated measure ANOVA associated with the LSD *post-hoc* test allowing multiple paired comparisons. In the case of a non-normal distribution, we used a Friedman ANOVA. Statistical analysis was performed using Statistica and GraphPad Prism software, and all graphs were created with GraphPad Prism5 software.

## Results

### Maximum Voluntary Contraction of the Lower Limb

**Figure 2:**
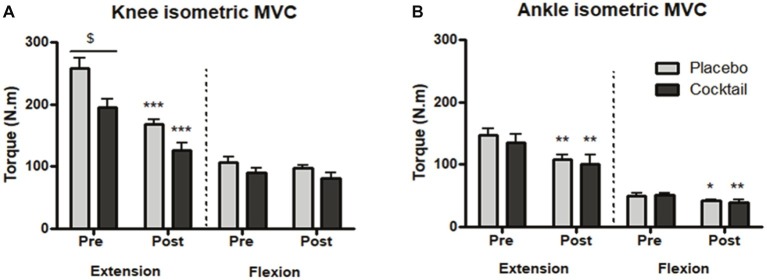
Isometric maximal voluntary contraction (MVC) torques before and after the HDBR experiment: torques (N.m) generated during isometric MVC in **(A)** knee and **(B)** ankle extension and flexion positions. Data bars represent means ± SEM. ^*^p < 0.05, ^**^p < 0.01, ^***^p < 0.001: difference between time condition (pre vs. post). ^$^*p* < 0.05: difference between groups (cocktail vs. placebo).

Isometric maximum voluntary contractions represented as torques (N.m) were measured before (pre) and after (post) 2 months of bedrest to evaluate the changes in muscle strength. Torques measured in the knee extension mode were significantly higher in the placebo group at the baseline point compared to the cocktail group (258 versus 195 N.m). However, torques measured in the knee extension mode significantly decreased after the bedrest period in both groups (−32% for the placebo group and −33% for the cocktail group; Figure 2A). Torques measured in the ankle extension and flexion modes were equal at baseline in the two groups and significantly decreased after bedrest in both groups (−16 and −25% for the placebo group and −22 and −25% for the cocktail group, in flexion and extension mode, respectively; Figure 2B).

These results demonstrated that 2 months of bedrest induced a significant loss of lower limb muscle strength, which was not prevented by cocktail supplementation.

### Myofiber Atrophy

**Figure 3:**
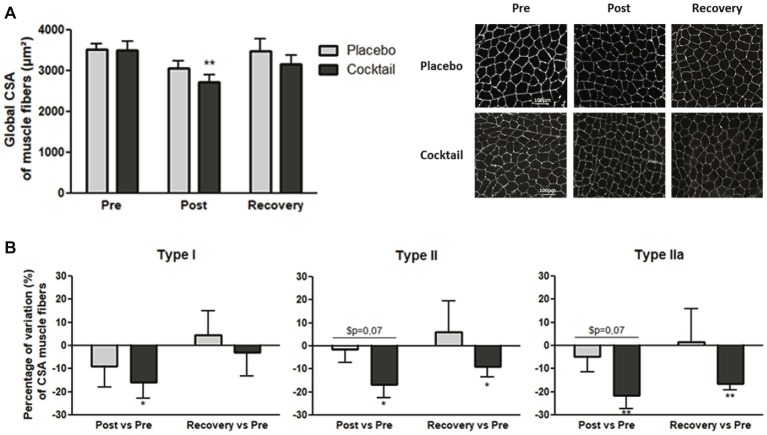
Cross-sectional areas (CSAs) of muscle fibers from vastus lateralis biopsies: **(A)** global CSA of muscle fibers (μm^2^) before (pre), after (post), and 10 days after the end (recovery) of 2-months HDBR; **(B)** variation of CSA of Type I, Type II, and Type IIa muscle fibers, between different time conditions. Data bars represent means ± SEM. ^*^p < 0.05, ^**^p < 0.01: difference between time condition. ^$^difference between group treatment (cocktail vs. placebo).

Global (all fiber types) and specific fiber type cross-sectional areas (CSAs) were identified from biopsies of pre, post, and recovery conditions. The cocktail group demonstrated a significant decrease of −22.5% in muscle fiber CSA after bedrest (from 3,511 μm^2^ before to 2,720 μm^2^ after bedrest, *p* < 0.01), while the placebo group showed a −11.7% non-significant decrease (from 3,525 μm^2^ before to 3,063 μm^2^ after bedrest, *p* = 0.08) (Figure 3A). Although the degree of atrophy was higher in the cocktail group compared to the placebo group, this difference was not statistically significant. Looking at the variation of CSA of each specific fiber type (Figure 3B), we observed that every type of muscle fiber was atrophied in the cocktail group after 2 months of bedrest (−16, −17, and − 21% for types I, II, and IIa, respectively). For the placebo, a non-significant decrease was observed in all muscle fiber types. After 10 days of recovery, type I fibers of the cocktail group reached the same size observed at baseline, while type II and IIa fibers were still smaller compared to baseline. The fast fiber types (types II and IIa) were more affected and were not recovered at 10 days after bedrest.

These results highlighted that the cocktail was not able to prevent skeletal muscle atrophy. Moreover, subjects that were supplemented with the cocktail were even more affected by myofiber atrophy, particularly type II and IIa muscle fiber atrophy.

### Changes in Myofiber Type Distribution

**Figure 4:**
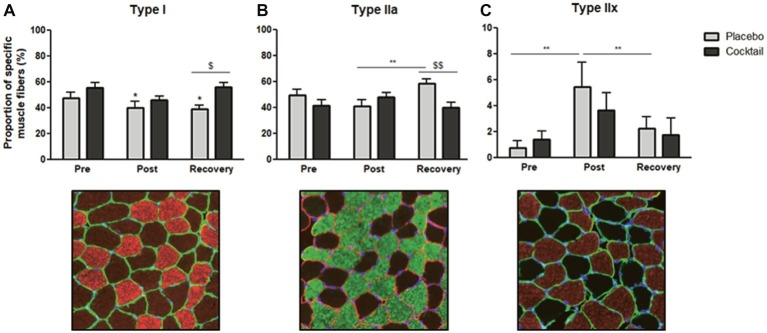
Distribution (%) of muscle fibers in *vastus lateralis* samples, in the different time-points and conditions of the HDBR experiment: proportion of **(A)** Type I, **(B)** Type IIa and **(C)** Type IIx muscle fibers before (pre), after (post), and 10 days after the end (recovery) of 2-months HDBR. Data bars represent means ± SEM. ^*^p < 0.05, ^**^p < 0.01: difference between time condition. ^$^*p* < 0.05, ^$$^*p* < 0.01: difference between group treatment (cocktail vs. placebo).

At baseline, the proportions of fibers expressing MyHC I, MyHC IIa, and MyHC IIx were equal in both groups. In the placebo group, 2 months of bedrest induced a significant decrease in the percentage of fibers expressing MyHC I (48 versus 40%; Figure 4A). The percentage of fibers expressing MyHC I was still significantly lower after 10 days of recovery compared to baseline. In the cocktail group, no changes in the proportion of type I fibers were observed after the bedrest period compared to baseline. However, we observed a higher proportion of type I fibers at the recovery point compared to the placebo group (56 versus 39%).

In both groups, no changes in the proportion of type IIa fibers were observed after the bed rest period compared to baseline (Figure 4B). However, in the placebo group, the proportion of fibers expressing MyHC IIa was significantly higher after 10 days of recovery compared to the end of the bedrest (post) (59 versus 41%) and compared to the values for the cocktail group (59 versus 40%).

Focusing on the fibers expressing MyHC IIx, we observed a dramatic increase in their proportion in the placebo group between the pre- and post-treatment conditions (0.8 versus 5.5%), which returned to basal values after a short recovery (Figure 4C). Conversely, in the placebo group, no changes were observed for the cocktail group during all experiments, which suggests a possible protective effect of the cocktail in the expression of IIx fibers.

The results indicate that prolonged inactivity modulates muscle typology, reducing some of the slow fibers (type I) for the benefit of fibers expressing MyHC IIx and that the cocktail is able to prevent this classical parameter observed in unloading and microgravity situations.

### Oxidative Stress Parameters

**Figure 5:**
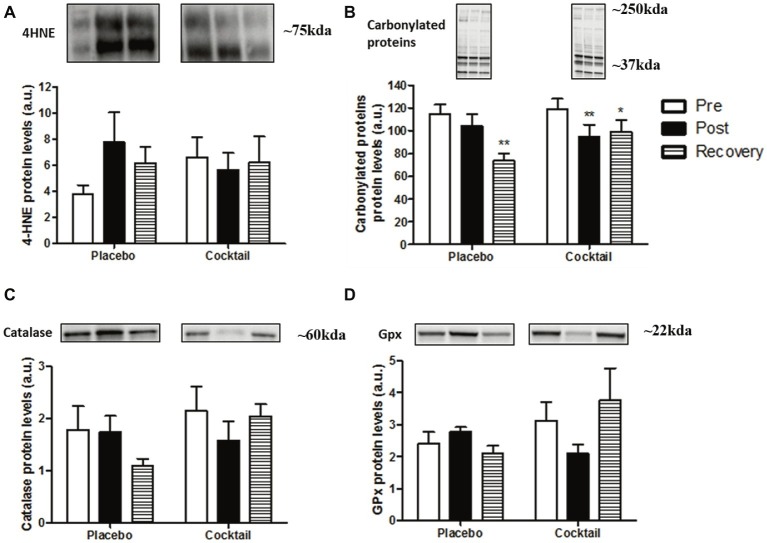
Oxidative stress parameters in the different time-points and conditions of the HDBR experiment: **(A)** determination of oxidative damage to lipids using 4-hydroxynonenal and **(B)** carbonylated protein levels. Protein content of antioxidant enzymes, **(C)** glutathione peroxidase, and **(D)** catalase. Data bars represent means ± SEM. ^*^p < 0.05, ^**^p < 0.01: difference between time condition.

To evaluate macromolecule ROS-induced damage, we analyzed the levels of 4-hydroxynonenal (4-HNE), a marker of lipid peroxidation, and the levels of carbonylated proteins. No significant change was observed during the experiment in either group for 4-HNE levels. However, 4-HNE levels tended to be increased in placebo subjects after bedrest but not in the cocktail group (Figure 5A). Levels of carbonylated proteins did not change after bed rest in the placebo group, while there was a significant decrease in the cocktail group (−19.7%, *p* < 0.01). In both groups, carbonylated protein levels were significantly decreased after 10 days of recovery compared to baseline (Figure 5B).

To explain oxidative damage, we examined the expression of endogen antioxidant defense and measured glutathione peroxidase and catalase protein levels in muscles. No significant differences between groups or time conditions were found (Figures 5C,D). However, we observed the same trend for the two enzymes at the recovery y point: the placebo group had lower contents compared to the cocktail group.

These results underline a potential protector effect of cocktail supplementation regarding the oxidative damage in muscle, without impairment of antioxidant enzyme expression after 2 months of hypoactivity and 10 days of recovery.

### Oxidative Metabolism Markers

**Figure 6:**
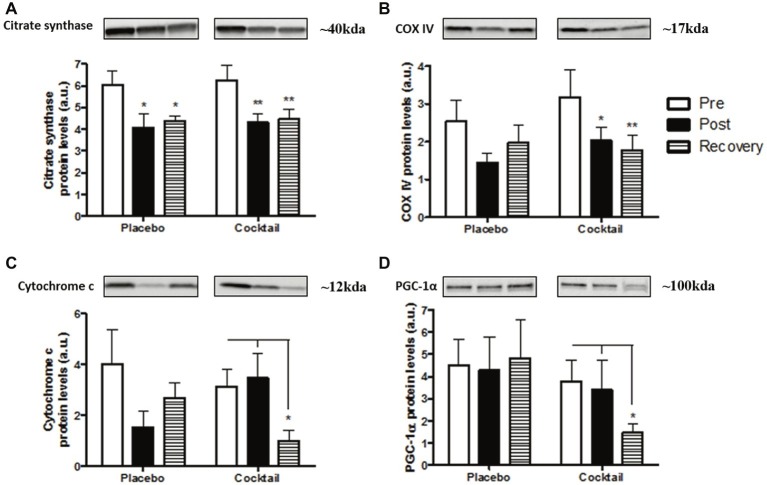
Oxidative metabolism parameters in the different time-points and conditions of the HDBR experiment: determination of **(A)** citrate synthase; **(B)** COX IV; **(C)** cytochrome c, and **(D)** PGC1-α protein levels. Data bars represent means ± SEM. ^*^p < 0.05, ^**^p < 0.01: difference between time condition.

To characterize changes in oxidative metabolism, we analyzed various mitochondrial parameters from vastus lateralis samples. In the cocktail group, two important markers of mitochondrial content, citrate synthase and COX IV, were significantly decreased after bedrest (pre versus post values) and were maintained at the same level after 10 days of recovery (Figures 6A,B). For placebo subjects, only citrate synthase protein levels were significantly decreased after bedrest (Figure 6A), while a trend to lower levels was observed for COX IV (*p* = 0.12) and cytochrome c (*p* = 0.16), (Figures 6B,C). However, cytochrome c protein levels were dramatically decreased after 10 days of recovery in the cocktail group, while values in the placebo group tended to increase (Figure 6C). PGC-1α protein levels, the master regulator of mitochondrial biogenesis, were also measured. No changes were observed after the bedrest period in either group. However, the cocktail group showed a drastic significant decrease in PGC-1α protein levels after 10 days of recovery (−61%; Figure 6D), while no changes were observed in the placebo group. Lower levels of both proteins were found in cocktail group samples at the recovery point and attested to a rapid loss of oxidative metabolism efficacy (Figures 6C,D).

### Protein Balance Pathways

**Figure 7:**
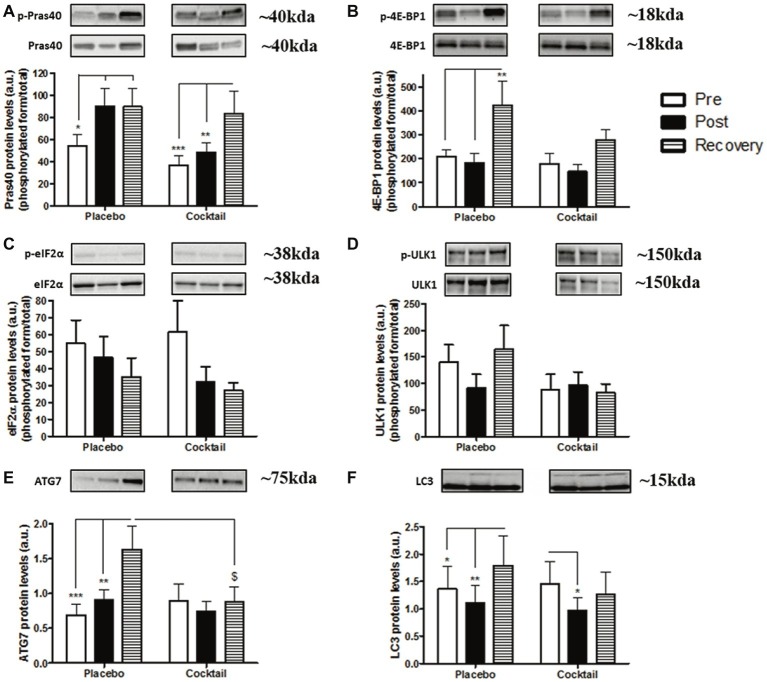
Protein balance parameters in the different time-points and conditions of the HDBR experiment: determination of synthesis pathway markers, **(A)** Pras40; **(B)** 4E-BP1; **(C)** eIF2α and degradation pathway markers **(D)** ULK1; **(E)** ATG7; and **(F)** LC3 protein levels. Data bars represent means ± SEM. ^*^p < 0.05, ^**^p < 0.01: difference between time condition. ^$^*p* < 0.05: difference between groups (cocktail vs. placebo) for the same time condition.

We evaluated changes in protein synthesis and degradation pathways under different conditions. We first analyzed markers of the main protein synthesis pathway regulated by mTOR. Levels of phosphorylated Pras40, whose phosphorylation by Akt permits the activation of mTORC1, were increased for the placebo group at the end of bedrest (+66%, *p* < 0.05; post) and 10 days after it (recovery) with respect to the pre values (+64%, *p* < 0.05). For the cocktail group, no changes were observed after bedrest, while a significant increase was observed at the recovery point compared to baseline (+127%, *p* < 0.001) and after bed rest (+72%, *p* < 0.01) (Figure 7A). This result demonstrates that mTORC1 is possibly more activated after the immobilization period. The result of phosphorylated 4E-BP1, an activator of elongation processes directly phosphorylated by mTORC1, showed no difference after bed rest in either group. However, a dramatic increase of phosphorylated 4E-BP1 was observed in the placebo group after 10 days of recovery compared to baseline and after bed rest (*p* < 0.01 in both cases). Such an increase was not observed in the cocktail group (Figure 7B). The phosphorylation of eIF2α, which is a subunit of the eIF2 initiation factor, has an inhibitory action on protein translation. Its content tended to decrease after bedrest in both groups, which means a lower inhibition of protein translation phenomenon after bedrest (Figure 7C). These results support the idea that after a long hypoactivity period, the protein synthesis pathway regulated by mTOR is impaired, while the recovery period is associated with the activation of this latter. However, activation during recovery appears to be impaired by the cocktail.

We studied three markers of the autophagic pathway, one of the main systems responsible for skeletal muscle mass regulation. ULK1, which is phosphorylated and inhibited by mTOR, is considered the first initiator of the autophagic process. No significant differences were observed between conditions and time (Figure 7D). However, the protein levels of two actors in autophagosome formation (latter stage of autophagy), ATG7 and LC3 (ratio of LC3 II/LC3 I), were higher at the recovery point with respect to pre- and post-treatment conditions in the placebo group (Figures 7E,F). These results suggest that the recovery period is associated with the activation of the autophagic process, which is inhibited by our cocktail.

## Discussion

The present study aimed to evaluate the effects of a dietary cocktail supplementation during 60 days of HDBR. It was hypothesized that the antioxidant and anti-inflammatory components of the cocktail would have a protective/preventive action against hypoactivity-induced muscle deconditioning.

After 2 months of HDBR, all subjects presented muscle deconditioning characterized at the functional level by a loss of muscle strength. Isometric MVC of quadriceps and triceps surae muscle groups were decreased in placebo (−32 and −21%, respectively) and cocktail subjects (−33 and −26%, respectively). Previous studies using the same protocol duration (60 days), longer duration (90 days) or shorter duration (28 days) found a loss of lower limb muscle strength (Zachwieja et al., 1999; Alkner and Tesch, 2004; Trappe et al., 2007; Kramer et al., 2017). Recently, using the dry immersion model of simulated microgravity, our research group showed that a few days of hypoactivity induced a decrease of 9% of MVC (Demangel et al., 2017). Concerning this first functional parameter, our results show no effects of cocktail supplementation on muscle strength loss.

It is known that inactivity-induced loss of force is the consequence of a variety of factors: atrophy of muscle cells (Chopard et al., 2009a,b), innervation deficiency (Kawakami et al., 2001), dysregulation of intracellular calcium machinery (Thom et al., 2001), and fatty infiltrations (Delmonico et al., 2009; Pagano et al., 2018). Among these elements, amyotrophy plays a predominant role. In our study, cocktail supplementation did not prevent muscle atrophy and even accentuated the decrease in muscle fiber CSA after 60 days of HDBR compared to the placebo group. Global CSA (−22%), as well as type II (−16% both) and type IIa (−21%), muscle fibers of supplemented subjects exhibited significant atrophy after bedrest. More particularly in this group, fast muscle fibers (type II) were more affected than slow fibers (type I). In humans, the literature has described that fast fibers may be more susceptible to microgravity-induced atrophy than oxidative fibers (Edgerton et al., 1995; Widrick et al., 1999; Fitts et al., 2000). Muscle deconditioning is also characterized by changes in muscle fiber typology. In the present study, all subjects exhibited the same fiber type distribution before the experiment. However, 10 days after the end of HDBR (recovery), supplemented subjects presented a higher percentage of type I fibers to the detriment of type IIa (56% of type I and 40% of type IIa) compared to placebo subjects (39% of type I and 58% of type IIa). In the placebo group, the increase of IIx fiber proportion (+718%) after bedrest traduced the contractile properties modification induced by hypoactivity. Interestingly, cocktail supplementation seems to limit the expression of IIx fibers classically observed during muscle deconditioning. The same tendency was also described in human experiments. For example, Edgerton et al. (1995) reported a decrease from 48 to 40% of type I fibers after 11 days of spaceflight. In our study, placebo subjects exhibited the same rate of reduction (47.8% in pre vs. 40% in post condition). We can establish a comparison with the results of Desaphy et al. (2010) who evaluated the effects of antioxidant supplementation during a hindlimb suspension period in mice. The administration of Trolox, a vitamin E analog, was unable to protect disused muscles from atrophy but partially prevented the MHC isoform redistribution in soleus muscles of the supplemented animals. Regarding the loss of muscle mass, our findings are contrary to the initial hypothesis, i.e., a potential supplementation’s protection against muscle wasting. Interestingly, although atrophy levels were higher in the cocktail group, the magnitude of strength loss was similar in both groups. This may indicate that antioxidant/anti-inflammatory supplementation would have reduced the relative part of other factors responsible for strength diminution. Extensive investigations are necessary to evaluate the role of every different factor in muscle strength loss contribution.

Situations of reduced activity are known to be sources of inflammation and cellular RONS production (Margaritis et al., 2009; Powers et al., 2011b). In the present study, we decided not to focus on inflammatory processes because of the lack of variation observed in the main marker of inflammation TNF-α in vastus lateralis (data not shown). In skeletal muscles, RONS induced damage to macromolecules such as lipids and proteins. Kondo et al. (1991) was the first study hypothesizing the contribution of redox disturbances to muscle atrophy and reported that prolonged inactivity was associated with high levels of lipid peroxidation in rats. Here, after 60 days of HDBR, the 4-HNE marker representing lipid peroxidation tended to increase in the placebo group, whereas lower levels of carbonylated proteins were observed in the supplemented group. These results proved that supplementation appears to protect against RONS-induced damage. Nevertheless, these positive effects on macromolecules did not provide better protection against muscle atrophy. To explain this, we hypothesize that the cocktail could be too rich in antioxidants, and it would abolish the beneficial role of RONS. Indeed, minimal amounts of these molecules are necessary for the healthy function of various physiological processes. If RONS are present in large quantities, oxidative stress occurs, whereas the absence of RONS induces “reductive stress” (Narasimhan and Rajasekaran, 2015).

Our results highlight that an imbalance in favor of antioxidant molecules during a prolonged inactivity period could accentuate skeletal muscle wasting. This finding underlines the complexity of redox balance mechanisms and demonstrates that physiological amounts of RONS are essential to activate molecular pathways and preserve positive adaptations. Indeed, a number of studies, particularly in the exercise training context, described that over-supplementation with exogenous antioxidants impairs the molecular signaling required for cellular adaptations (Chang et al., 2007; Gomez-Cabrera et al., 2008; Ristow et al., 2009; Merry and Ristow, 2016).

On the other hand, a body of evidence revealed that prolonged muscle inactivity induces oxidative capacity alteration and mitochondrial dysfunction, leading to activation of atrophic pathways (Hyatt et al., 2019). The major regulator of mitochondrial biogenesis, PGC-1α, and various key mitochondrial proteins is known to be downregulated during muscle inactivity (Chen et al., 2007; Kang et al., 2013). Other studies also demonstrated that hypoactivity leads to oxidative metabolism gene downregulation in animals and in women during bedrest (Chopard et al., 2009a,b; Brocca et al., 2010). In our study, lower protein levels of citrate synthase and COX IV after HDBR indicated a decrease in the mitochondrial content in the skeletal muscles of all subjects. Moreover, 10 days of recovery were not sufficient to recover basal values. Similar results were observed in animals after 2 weeks of immobilization followed by 5 days of recovery (Kang et al., 2013). In addition, the drop of PGC-1α and cytochrome c levels in the recovery point was only observed for the cocktail group. This suggests that the oxidative metabolism of supplemented subjects was more affected than that of the placebo subjects. The sudden stop of antioxidant supplementation after 60 days certainly disturbed the molecular pathways involved in mitochondrial dynamics. This idea is in agreement with the study of Gomez-Cabrera et al. (2012), which indicates that antioxidant supplements were responsible for limited mitochondrial adaptations during aerobic training.

Research has long described that skeletal muscle hypoactivity causes a dysregulation of signaling pathways involved in muscle mass maintenance. Alterations of protein balance mechanisms occur at an early stage, from the first day of hypoactivity, and then tend to stabilize (Kawashima et al., 2004). Due to the long duration of our HDBR protocol, we especially wanted to evaluate the modulation of synthesis and degradation pathways in the days following remobilization. Analyses performed in the recovery point provided information regarding molecular dynamics occurring after long-term inactivity and envisage the rate of muscle recuperation. The PI3K-Akt-mTOR axis is the major pathway activating protein synthesis in skeletal muscle. Here, the elevation of Pras40 protein levels, whose phosphorylation by Akt activates the mTORC1 complex, and the increase of 4E-BP1 levels suggest an activation of the main synthesis pathway in the recovery period. This idea is strengthened by the reduction in eIF2α contents at the same time, knowing that its phosphorylation has an inhibitory action on protein translation. Data in the literature indicate that elevated RONS production can inhibit Akt/mTORC1 signaling (Powers et al., 2016). However, in our study, all subjects, supplemented or not, demonstrated the same dynamic. This indicates that the cocktail does not reduce muscle wasting recovery processes. Our results illustrate the same insight as that described by various studies focusing on antioxidant supplementation and strength training adaptations. All of these studies highlight that additional antioxidants (Vitamin C and/or E) may hamper the optimum activation of important hypertrophic pathways (Makanae et al., 2013; Paulsen et al., 2014; Bjørnsen et al., 2016; Dutra et al., 2018).

We also aimed to investigate the evolution of autophagy parameters. Indeed, autophagy is a major proteolytic pathway whose activation during inactivity accentuates muscle wasting. Numerous reports have evoked the potential of RONS to accelerate protein breakdown *via* this pathway (Navarro-Yepes et al., 2014; Pajares et al., 2015). The mechanisms by which RONS promote autophagy remain unclear, but they could increase ULK1 activity, the initiator of autophagy processes, through the downregulation of mTORC1. In the present study, although no significant differences in ULK1 activation were observed between conditions, and the levels of ATG7 and LC3II/LC3I ratio were significantly higher at recovery points compared with pre- and post-conditions for placebo subjects. These results indicate an increase in key autophagy components after remobilization, but this evolution was not observed in the supplemented subjects. Moreover, the absence of a difference between post- and pre-conditions underlined that autophagy flux was not overstimulated after 2 months of inactivity. If muscle wasting is partly due to an increase of protein degradation pathways, this was not perceptible after such a long time and was certainly detectable in the first days of immobilization. Here, cocktail supplementation seems to abolish autophagy dynamics in skeletal muscle remobilization. At present, there is still a lack of literature available regarding antioxidants and muscle autophagy pathways, but we infer that a dysregulation in favor of pro-oxidants may blunt some molecular mechanisms responsible for the control and the recovery of muscle mass. Further investigations are needed to distinguish the role of exogenous intake of antioxidants on these pathways during immobilization, especially its impact on subsequent recovery.

Previous cited studies showing signaling pathways disturbance using antioxidants were done with direct RONS scavengers (Vitamin C and E) with relative high doses. Similar results have been recently obtained during aerobic adaptations to cycling training in humans with epicatechin, a flavonol which can modulate superoxide dismutase and glutathione peroxidase and activate antioxidant gene such as PGC-1α (Schwarz et al., 2018). In this study, the dose of epicatechin was very low compared to our study since only 200 mg per day were used. Moreover, it is important to underline that in our present study, the dose of the cocktail’s components was the same for each individual, independently of their specific needs. These results show that regardless of the type of antioxidant used alone or combined, if the dose is not personalized, the effects may not be beneficial. Recently, the group of Paschalis et al. published two interesting studies which highlight the importance of adapting antioxidant supplementation to the personalized requirements of the subjects. In these studies, vitamin C and N-acetyl-cysteine (a glutathione precursor) were used alone with individualized doses in regard to blood vitamin C and glutathione levels, respectively. Beneficial effects (increase of maximal oxygen uptake and/or maximal power) were obtained only in people with low blood vitamin C or glutathione levels. These results showed that the effectiveness of antioxidant supplementation largely depends on the redox status of the subjects receiving the treatment (Paschalis et al., 2016, 2018). In our cocktail, vitamin E acts as a RONS scavenger without a specific action. Some studies using Allopurinol, an inhibitor of xanthine oxidase (a major RONS sources during muscle unloading), showed limitation of muscle atrophy in hindlimb suspended rodents and in humans with ankle sprain (Derbré et al., 2014; Ferrando et al., 2018). Compared to our cocktail which does not act on a specific RONS source or RONS family involved during muscle unloading, these studies highlight the importance of choosing an antioxidant strategy with a specific action corresponding to the need of the subject situation (inactivity, aging, microgravity,…). Finally, in the future, antioxidant strategies should be personalized based on the redox status of each subject and should be chosen in function of their action on RONS source and/or RONS family.

## Conclusions

In aerospace applications, understanding muscle deconditioning mechanisms induced by microgravity environments constitutes an essential issue. The results obtained through these studies are also validated in the context of muscle deconditioning prevention, for clinical hospitalization, immobilization post injuries, or more generally chronic hypoactivity. Experimental models provide the opportunity to test countermeasures and strategies and to evaluate their effects on disuse-induced atrophy. Physical exercise is the main intervention that demonstrated positive effects (Fitts et al., 2010; Gao et al., 2018), and some studies combined it with protein or growth factor supplementation (Allen et al., 1997; Brooks et al., 2008). Despite its benefits, exercise training seems insufficient to limit muscle wasting in prolonged hypoactivity periods, and research of an efficient and feasible countermeasure including nutritional intervention is still in progress. The present study was conducted to evaluate the effects of a cocktail enriched in antioxidant/anti-inflammatory molecules in a 2-month HDBR experiment. This countermeasure was expected to limit the effects of muscle deconditioning, but our results clearly demonstrate the ineffectiveness of supplementation in the prevention of muscle mass and strength loss. Moreover, data regarding muscle molecular mechanisms highlight an alteration of recovery processes in the supplemented subjects.

These results can be explained by an inhibition of the beneficial adaptations induced by the presence of RONS and illustrate the necessity of pro-oxidant molecules during long-term inactivity to maintain a certain level of muscle function.

Our conclusions underline the complexity of redox mechanisms and raise interrogations regarding the appropriate nutritional intervention to fight against muscle deconditioning.

## Data Availability Statement

The datasets generated for this study are available on request to the corresponding author.

## Ethics Statement

The studies involving human participants were reviewed and approved by CPP Sud-Ouest et Outre-Mer I, France, number ID RCB: 2016-A00401–50. The patients/participants provided their written informed consent to participate in this study.

## Author Contributions

CA-C, GP, TF, TB, SB, and AC helped in conceptualization and methodology. CA-C, GP, TF, TB, and AC helped in formal analysis, writing original draft preparation, and visualization. CA-C, TB, TF, RR, RD, PD, and AP helped in investigation. DB, MS, M-CG-C, and JV helped in finding resources. CA-C, TF, TB, and GP helped in data curation. CA-C, TB, AC, SB, BJ, MS, M-CG-C, and JV helped in supervision. CA-C, GP, TB, and AC helped in project administration and writing—review and editing. GP, BJ, DB, TB, and AC helped in funding acquisition. All authors read and approved the final manuscript.

### Conflict of Interest

The authors declare that the research was conducted in the absence of any commercial or financial relationships that could be construed as a potential conflict of interest.
